# Editorial: Reproductive microbiome and its interplay with the environment

**DOI:** 10.3389/fendo.2024.1470608

**Published:** 2024-08-15

**Authors:** Federica Cariati, Alessandro Conforti, Sandro C. Esteves, Carlo Alviggi

**Affiliations:** ^1^ Department of Public Health, University of Naples Federico II, Naples, Italy; ^2^ Department of Neuroscience, Reproductive Science and Odontostomatology, University of Naples Federico II, Naples, Italy; ^3^ ANDROFERT, Andrology and Human Reproduction Clinic, Campinas, Brazil; ^4^ Department of Surgery (Division of Urology), State University of Campinas (UNICAMP), Campinas, Brazil; ^5^ Department of Clinical Medicine, Faculty of Health, Aarhus University, Aarhus, Denmark

**Keywords:** microbiota, environment, male/female fertility, semen, testicle reproduction, uterus, vagina, assisted reproductive techniques

The human microbiome plays a vital role in maintaining a balance of physiologic functions. By expressing bacterial genes, it integrates and regulates human metabolism. However, the microbiome can change according to lifestyle and environmental factors. It is also linked to certain pathological states, the so-called “diseases of progress”, especially prevalent in high-income, developed countries. The presence of microorganisms in the reproductive tract is well documented, and it has been demonstrated that the resident microflora interacts with the biological mechanisms responsible for conception and implantation. Yet, there is limited data on the impact of the seminal microbiome on the female tract during natural conception or assisted reproductive techniques. In contrast, the vaginal and endometrial microbiota are suggested to play a role in fertilization.

Thanks to advancements in metagenomics, it is now possible to sequence 16S rRNA, a specific RNA gene beneficial for ribosome production and protein synthesis in bacteria. Studying the microbiome/microbiota in various human fluids from molecular and biochemical perspectives allows us to hypothesize its use as a biomarker in different physiological and pathological conditions. However, more research is needed to explore potential clinical implications and therapeutic interventions, such as microbial therapeutics and lifestyle adaptations. This Research Topic includes six articles investigating the role of microbiota or microbiome in reproductive disorders and infertility, as well as the potential effects of probiotic treatments.

Wang et al. examined the correlation between intestinal bacteria and primary ovarian insufficiency (POI) using data from human and animal models. They conducted the first large-scale Mendelian randomization study to assess the causal relationship between intestinal bacteria and POI, using genetic data from the MiBioGen and FinnGen consortia. The results revealed a beneficial causal link between Eubacterium hallii, Eubacterium ventriosum, and POI and a detrimental link between Testinibacter and Terrisporobacter and POI.

Zizolfi et al. presented a narrative review on the relationship between microbiota and endometriosis, revealing a higher abundance of pathogenic species in women with endometriosis. This disrupts immune function and stimulates inflammatory factors. Additionally, increased microbial richness and chronic inflammation seem to correlate with adenomyosis. The review discusses studies on chronic pain related to lower gut microbiota diversity, suggesting a potential influence on acyloxy acyl hydrolase levels, which play a role in modulating pelvic pain. Probiotics could provide benefits by regulating visceral inflammatory pain.

Jepsen et al. investigated the use of lactobacilli-loaded vaginal capsules to reestablish vaginal eubiosis in 74 women with low Lactobacillus or high disrupting bacteria levels before fertility treatment. This randomized, double-blinded, placebo-controlled study found no significant difference in vaginal microbiota improvement between women who received one vaginal capsule daily containing Lactobacillus (L. gasseri and L. rhamnosusper) for ten days and those who received a placebo. No adverse effects were reported in the treated group. However, the study showed a spontaneous improvement in unfavorable vaginal microbiota in 34.2% of patients within a period of one to three months, suggesting a potential benefit in postponing further IVF treatment to await vaginal eubiosis.

Blancafort et al. noted the challenges of studying the vaginal and endometrial microbiome and probiotic therapies due to heterogeneity in variables like diagnostic methods, strains used, and delivery methods. Their systematic review on probiotics in the female genital tract microbiota highlighted that assessing the beneficial effects of the *Lactobacillus* genus on fertility is complex, as different species have different effects. The delivery method, whether oral or vaginal, also presents confounding factors. The study suggests that exogenous Lactobacillus may struggle to colonize and replicate in the female tract epithelium.

Cariati et al. demonstrated that endometrial microbiota generates significant interest in infertile patients. Their study, which analyzed endometrial fluid after embryo transfer in women undergoing IVF, found that over 70% of patients tested positive for one or more microbes using culturomics analysis. *Lactobacillus* species were positively correlated with pregnancy rates, while Staphylococcus subspecies (spp.) and Enterobacteriaceae negatively impacted implantation rates. Culturomics using a double-lumen catheter set for embryo transfer was shown to be a reliable method to minimize vaginal contamination and detect pathogen growth. These results align with the hypothesis of Jepsen et al. to analyze the microbiota before IVF treatment and wait for a permissive microbial community for ongoing pregnancy.

Neto et al. reviewed the importance of studying the seminal microbiome before an IVF cycle, noting its discernible effects on reproductive outcomes. They found that sexually active couples shared 56% of predominant genera, and among couples with positive cultures in both partners, 61% shared at least one genital pathogen. However, the composition of the seminal microbiome, related to semen quality in terms of sperm concentration, motility, morphology, and DNA integrity, remains controversial. The seminal microbiome, which represents a mixture of the microbiomes of the testicular, epididymal, prostatic, seminal vesicular, and urethral microbiomes, is influenced by factors such as a high-fat diet that might lead to gut microbiota dysbiosis, ultimately affecting spermatogenesis and sperm motility. The authors also noted that sexually transmitted infections were associated with decreased semen microbiome diversity and richness.

This Research Topic is of broad interest. We are grateful to the Editorial staff of Frontiers in Endocrinology (Reproduction) for their support. This Topic is recommended to clinicians managing infertile couples, gynecologists specializing in endometriosis and POI, andrologists, and reproductive urologists. We hope that this Research Topic also encourages further research to elucidate the role of probiotics and best practices in sample collection. Additionally, establishing robust criteria to identify the “optimal” human microbiome profile is crucial for optimizing human fertility.

